# Echocardiographic assessment in arterial hypertension: a contemporary narrative review

**DOI:** 10.1186/s44156-026-00125-3

**Published:** 2026-06-29

**Authors:** Hafiz Naderi, Kelly Victor, Lynne Williams, Pauline A. Swift, Ian Wilkinson, Sanjeev Bhattacharyya

**Affiliations:** 1 William Harvey Research Institute, Queen Mary University of London, Charterhouse Square, London, EC1M 6BQ UK; 2 Barts Heart Centre, St Bartholomew’s Hospital, Barts Health NHS Trust, West Smithfield, London, EC1A 7BE UK; 3 Cleveland Clinic London, 33 Grosvenor Place, London, SW1X 7HY UK; 4 Department of Cardiology, Royal Papworth Hospital NHS Foundation Trust, Cambridge, CB2 0AY UK; 5 Renal Services, Epsom and St Helier University Hospitals NHS Foundation Trust, Carshalton, Surrey, SM5 1AA UK; 6 Clinical Pharmacology Unit, Cambridge University Hospitals NHS Foundation Trust, Cambridge, CB2 0QQ UK; 7 Department of Experimental Medicine and Immunotherapeutics, University of Cambridge, Cambridge, CB2 0BB UK

**Keywords:** Blood pressure, Echocardiography, Hypertension, Hypertensive heart disease, Left ventricular hypertrophy

## Abstract

Arterial hypertension remains the leading modifiable cause of cardiovascular morbidity and mortality resulting in characteristic changes in cardiac structure and function. Contemporary hypertension clinical guidelines increasingly emphasise prevention and early detection of hypertension-mediated organ damage, yet they differ in how blood pressure is categorised and when further investigation is recommended. Within this prevention-focused landscape and alongside a broader cardio-renal-metabolic framework, this review provides a practical overview on the role of echocardiography in arterial hypertension by integrating contemporary hypertension guidelines with imaging consensus documents. We outline when echocardiography is most likely to add value in hypertension and review echocardiographic assessment of left ventricular structure and geometry, systolic and diastolic function and filling pressures, atrial remodelling, right ventricular assessment, valvular heart disease and the aorta. Practical indications for scanning and transoesophageal echocardiography considerations are discussed. Finally, we highlight future directions including prevention-focused pathways, scalable echocardiography strategies, routine inclusion of contemporaneous blood pressure in reports, artificial intelligence-enabled quantification and selective multimodality imaging within specialist hypertension services.

## Introduction

Arterial hypertension is the strongest modifiable risk factor for cardiovascular disease worldwide, contributing substantially to the development of heart failure, ischaemic heart disease, stroke, chronic kidney disease and premature mortality [1]. Despite advances in pharmacological therapy and public health initiatives, blood pressure (BP) control remains suboptimal in many populations, and cardiovascular risk often accumulates silently over years before the onset of clinical disease [2]. In parallel, the accelerating obesity crisis has become central to contemporary preventive cardiology. As healthcare systems increasingly shift towards prevention-focused strategies, there is growing emphasis on addressing upstream drivers within a broader cardio-renal-metabolic framework, recognising the shared pathophysiological drivers, and reinforcing the importance of early detection of hypertension-mediated organ damage (HMOD) to refine risk stratification and guide timely intervention [3–5].

This shift has important implications for cardiac imaging. Contemporary guidelines differ in how BP is categorised and when additional investigation is recommended. The 2024 European Society of Cardiology (ESC) guideline introduces the concept of elevated BP at lower systolic thresholds, reflecting a prevention-oriented approach earlier in the disease trajectory, whereas the 2023 European Society of Hypertension (ESH) and the 2025 American Heart Association/American College of Cardiology (AHA/ACC) guidance differ in classification and the role of optional testing [6–8]. These differences influence how cardiac imaging is deployed across healthcare systems, particularly when the aim is not universal screening but selective identification of cardiac HMOD in patients in whom imaging may refine risk stratification or alter management.

Hypertensive heart disease (HHD) encompasses a spectrum of structural and functional cardiac changes resulting from chronic pressure overload, including left ventricular hypertrophy (LVH), altered ventricular geometry, diastolic dysfunction, atrial remodelling and progressive impairment of myocardial performance [9–11]. Transthoracic echocardiography (TTE) plays a central role in the evaluation of patients with arterial hypertension, providing comprehensive assessment of cardiac chamber size, wall thickness, ventricular mass, systolic and diastolic function, and haemodynamic consequences.[12, 13]. Beyond conventional parameters, advances in echocardiographic techniques, particularly myocardial strain imaging have enhanced the ability to detect early myocardial dysfunction before overt clinical manifestations arise. Furthermore, echocardiography enables evaluation of left atrial remodelling, right ventricular involvement, and secondary valvular abnormalities, all of which carry important prognostic implications in hypertensive populations [14–16]. Our aim in this review is to provide a practical, guidelines-anchored approach of how echocardiography should be used in arterial hypertension. Specifically, we focus on when to scan, what echocardiographic findings are most relevant to hypertension-mediated cardiac damage, and how echocardiography can refine cardiovascular risk beyond BP measurement alone within prevention-focused care pathways.

### Recommendations for echocardiography in contemporary hypertension clinical guidelines

Contemporary hypertension guidance increasingly emphasises a lifespan approach, with earlier identification of raised BP, confirmation with out-of-office measurements and risk-based treatment to prevent HMOD before symptoms occur [6–8]. In this context, TTE is positioned as a targeted investigation to detect and phenotype cardiac HMOD, rather than a universal screening test for all patients with raised BP. The 2024 ESC guideline recommends echocardiography when there are ECG abnormalities or clinical signs and symptoms suggesting LV dysfunction or structural heart disease, and notes that broader use in newly diagnosed hypertension may be considered where resources allow [7]. The 2023 ESH guideline similarly adopts a tiered approach to HMOD assessment (clinical evaluation and ECG as first-line), using cardiac imaging when it meaningfully reclassifies risk or influences treatment intensity, and supporting follow-up imaging in selected patients with established LVH [6]. The 2025 AHA/ACC guideline places echocardiography among ‘optional tests’ that may assist in detecting HMOD and refining risk stratification, alongside strong emphasis on accurate BP phenotyping with ambulatory or home monitoring [8].

Alongside these hypertension guidelines, modality-focused documents help translate recommendations into practical imaging pathways. The joint European Association of Cardiovascular Imaging (EACVI) and American Society of Echocardiography (ASE) recommendations (2015) emphasised that routine echocardiography in uncomplicated, asymptomatic hypertension is generally low value, while endorsing a trigger-based approach that prioritises scanning when suspicion of cardiac involvement is raised clinically or by ECG [13]. This was followed by the EACVI, ESC and ESH consensus statement (2017) on non-invasive cardiovascular imaging for subclinical target organ damage, which frames echocardiography within a broader multimodality approach, particularly in patients in whom the presence of organ damage would reclassify risk and influence treatment decisions [17]. These imaging-focused recommendations developed in an era shaped by large outcome trials that informed contemporary BP targets and risk-based strategies, including SPRINT and HOPE-3 [18, 19]. UK guidance from the British and Irish Hypertension Society (BIHS) highlights the importance of specialist evaluation in young-onset hypertension (aged < 40 years), resistant hypertension and suspected secondary causes, supporting targeted investigation rather than universal cardiac imaging [20, 21]. In parallel, the British Society of Echocardiography (BSE) triage resources for outpatient echocardiography requests and an accompanying primary care triage poster provide a nationally endorsed framework to prioritise scanning where there are symptoms, an abnormal ECG, suspected structural heart disease or when results are likely to alter management [22–24]. Consistent with the European and American recommendations, TTE in uncomplicated, asymptomatic hypertension with a normal ECG remains low yield, however, earlier imaging may be appropriate when symptoms, ECG abnormalities, resistant hypertension or suspected structural disease are present, and when findings are likely to influence management. In this review, pragmatic clinical scenarios for TTE assessment in hypertension, consistent with published guidelines and consensus recommendations are summarised in Table 1. In this table we summarise clinical scenarios in which TTE may be particularly useful in patients with hypertension, where the findings may identify cardiac HMOD, refine risk stratification, or influence management.

**Table 1:** Clinical scenarios for transthoracic echocardiographic assessment in arterial hypertension

Clinical scenario	Purpose of echocardiographic assessment
Abnormal ECG	Structural heart disease (e.g. LVH)
Suspected heart failure	RV/LV diastolic and systolic function
Suspected valvular heart disease	Valvular stenosis or regurgitation
Suspected coronary artery disease	Regional wall motion abnormalities, LV size and function
Suspected aortic coarctation	Assessment of distal arch and upper descending aorta
Refine cardiovascular risk	Prior to initiation of certain treatments (e.g. cardiotoxic chemotherapy)

ECG: electrocardiogram; LV: left ventricle; LVH: left ventricular hypertrophy; RV: right ventricle

### Pathophysiology of hypertensive heart disease

**Fig. 1:**
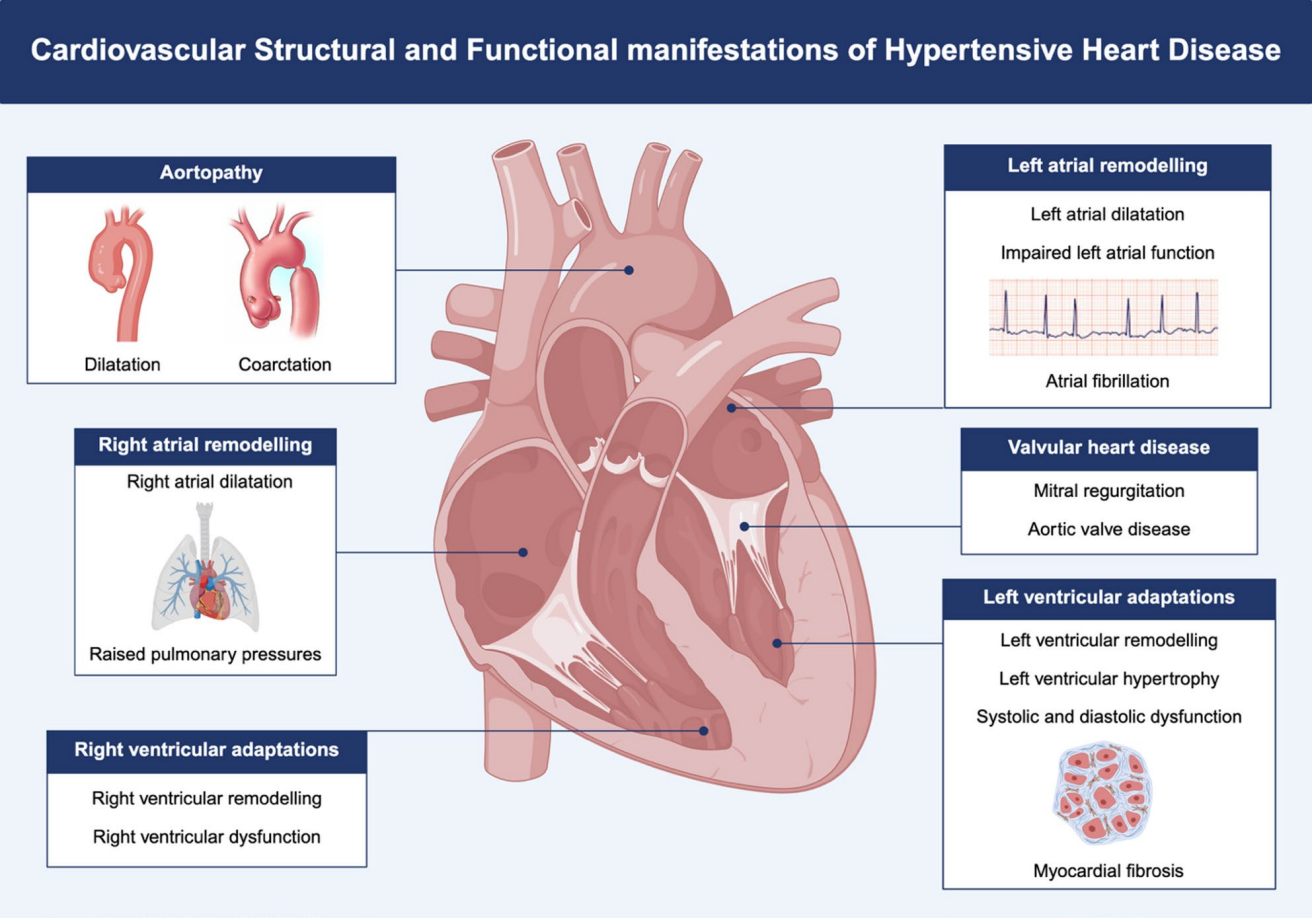
Cardiovascular structural and functional manifestations of hypertensive heart disease

Hypertensive heart disease (HHD) describes the structural and functional cardiac adaptations of sustained elevation in BP, summarised in Fig. 1. However, the extent to which these findings represent a distinct disease entity attributable to BP alone has been challenged, with recent commentaries arguing the term HHD may overstate direct causality which may reflect ageing and multimorbidity [25, 26]. Against this background, elevated BP remains an important contributor to adverse myocardial function via chronic pressure overload, progressive arterial stiffening and impaired ventricular–arterial coupling that increases central systolic and pulsatile load [10]. This mechanical burden drives compensatory myocardial hypertrophy and is accompanied by maladaptive pathways including neurohormonal activation, endothelial dysfunction, oxidative stress and inflammation, that promote extracellular matrix remodelling and reactive fibrosis, leading to increased stiffness, impaired relaxation and reduced coronary flow reserve [9]. Chronically raised filling pressures contribute to left atrial remodelling and predispose to atrial fibrillation, while downstream pulmonary venous hypertension may result in pulmonary hypertension and secondary right ventricular involvement, while concurrent chamber and annular remodelling may lead to functional valvular regurgitation and proximal aortic dilatation. Detailed echocardiographic characterisation of these domains is addressed in the dedicated sections that follow.

### Left ventricular structure and geometry

**Fig. 2:**
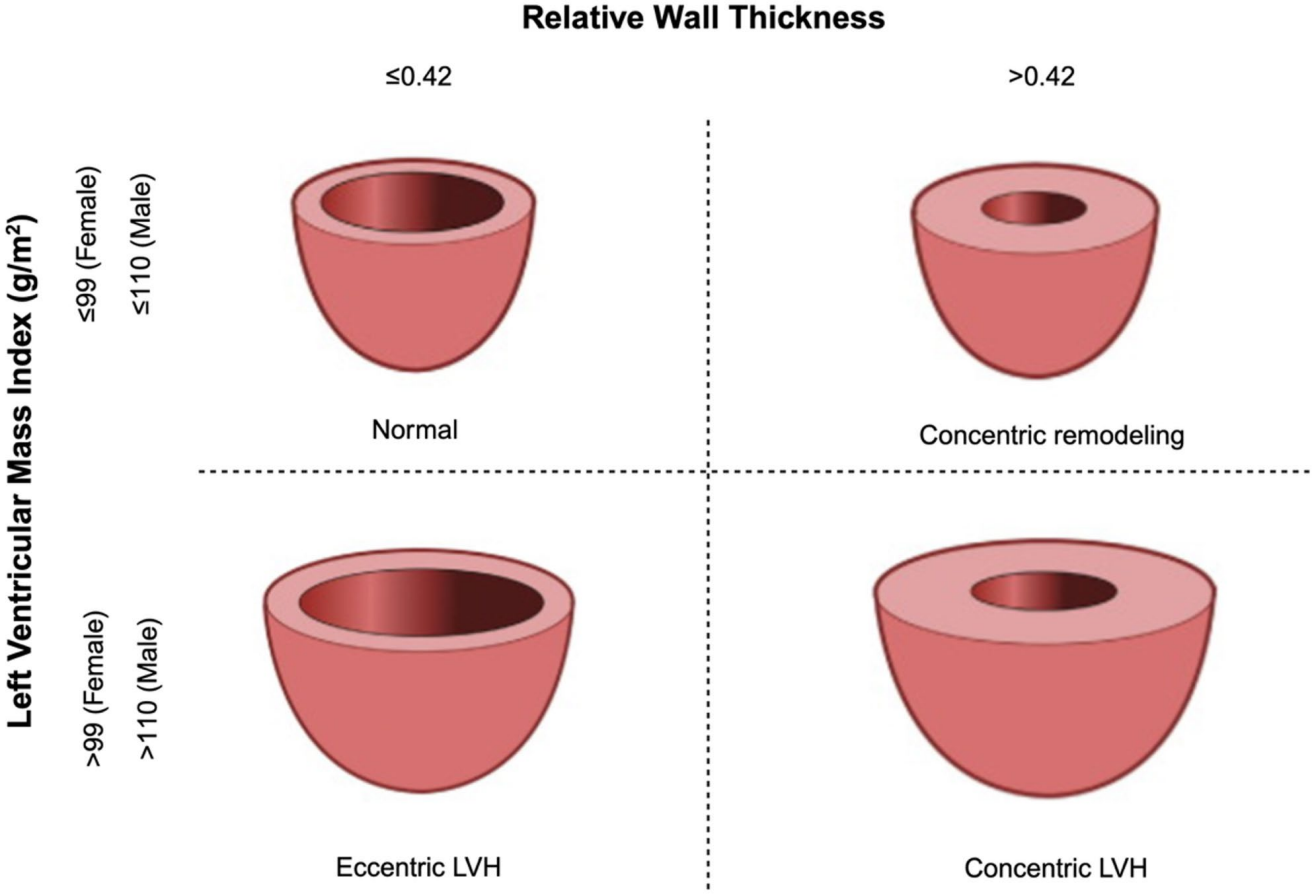
Patterns of left ventricular geometry and remodelling in hypertension

The physiological adaptations of the LV to sustained arterial hypertension have been recognised since the 1960s against the backdrop of the Pickering lectures [27, 28]. Chronically elevated afterload and wall stress drive cardiomyocyte hypertrophy, extracellular matrix remodelling and fibrosis [29]. Echocardiography has been central to describing the geometric patterns of LV adaptation using relative wall thickness (RWT) and LV mass [30]. Contemporary guidance categorises LV geometry as normal, concentric remodelling (normal LV mass with increased RWT), concentric hypertrophy (increased LV mass with increased RWT), or eccentric hypertrophy (increased LV mass with normal RWT), as illustrated in Fig. 2. Echocardiographic LV mass is an established independent predictor of cardiovascular events and mortality, supporting its role as a marker of cumulative BP exposure and underpinning the importance of accurate measurement of LV wall thickness and LV mass in phenotyping HHD [31, 32]. Compared with normal geometry, concentric remodelling and both hypertrophy patterns have been consistently associated with higher cardiovascular risk, with concentric and eccentric LVH representing the highest-risk phenotypes [33–36].

Importantly, increased wall thickness should not be assumed to be “hypertensive” in origin, particularly when the degree of thickening appears out of proportion to the severity or duration of BP elevation, when ECG voltage is either low or markedly increased, or when myocardial deformation is disproportionately abnormal [10]. In such settings, echocardiographic “red flags” include reduced global longitudinal strain with relative apical sparing (suggestive of cardiac amyloidosis) and very low mitral annular tissue Doppler e′ velocities (e.g. <5 cm/s), which may indicate an infiltrative or storage cardiomyopathy rather than uncomplicated hypertensive remodelling [37, 38]. When suspicion persists, multimodality imaging can clarify aetiology. Cardiac magnetic resonance (CMR) imaging provides reproducible assessment of LV mass and geometry and can characterise tissue using late gadolinium enhancement and mapping techniques (native T1/ECV), supporting differentiation between hypertensive remodelling, hypertrophic cardiomyopathy, amyloid infiltration and Fabry disease (including low native T1 in Fabry) [39, 40]. In suspected transthyretin amyloid cardiomyopathy, tracer scintigraphy (e.g. 99mTc-DPD) offers a validated non-invasive diagnostic pathway in the appropriate clinical context [41].

Distinct patterns of LV adaptation have also been described in specific hypertensive populations since the mid-1980s, including reports of a “hypertensive cardiomyopathy” phenotype in older adults [42, 43]. This phenotype is characterised by a relatively small LV cavity with concentric hypertrophy predominantly observed in female patients. A related and increasingly discussed finding is focal basal septal hypertrophy often termed a “septal bulge”, which may represent an early remodelling response in hypertension and which can be accentuated due to ageing with unfolding of the aorta, and requires careful interpretation and differentiation from hypertrophic cardiomyopathy when disproportionate, associated with dynamic obstruction, or accompanied by a suggestive family history [44–46]. Together, these observations reinforce that LV adaptation to hypertension exists on a spectrum, with important nuances related to sex, body size, exercise status and ethnicity that should be considered in clinical interpretation.

Antihypertensive therapy can lead to reverse remodelling, including reductions in LV wall thickness and LV mass and, in some patients, normalisation of adverse geometric patterns. Changes in LV mass are clinically relevant as demonstrated by Devereux and colleagues (2004) that reduction in LV mass during treatment was associated with improved prognosis, independent of baseline risk [47]. However, routine serial echocardiography for LVH regression is not recommended in uncomplicated, asymptomatic hypertension as measurement variability may obscure small but clinically meaningful change. Follow-up imaging is therefore best reserved for selected patients in whom results are likely to influence management (e.g. high-risk phenotypes, discordant clinical course, resistant hypertension, or reassessment of symptoms) [13, 7]. Where follow-up is performed, consistency of methodology and acquisition is essential, and 2D approaches may be preferable to linear methods for individual serial assessment. CMR provides the most reproducible quantification of LV mass and is well suited to longitudinal assessment in research and in selected clinical scenarios, while advances in 3D echocardiography may offer a pragmatic compromise by improving geometric fidelity and reproducibility compared with 2D linear techniques where image quality and expertise allow [48, 49].

### Left ventricular systolic function

**Fig. 3:**
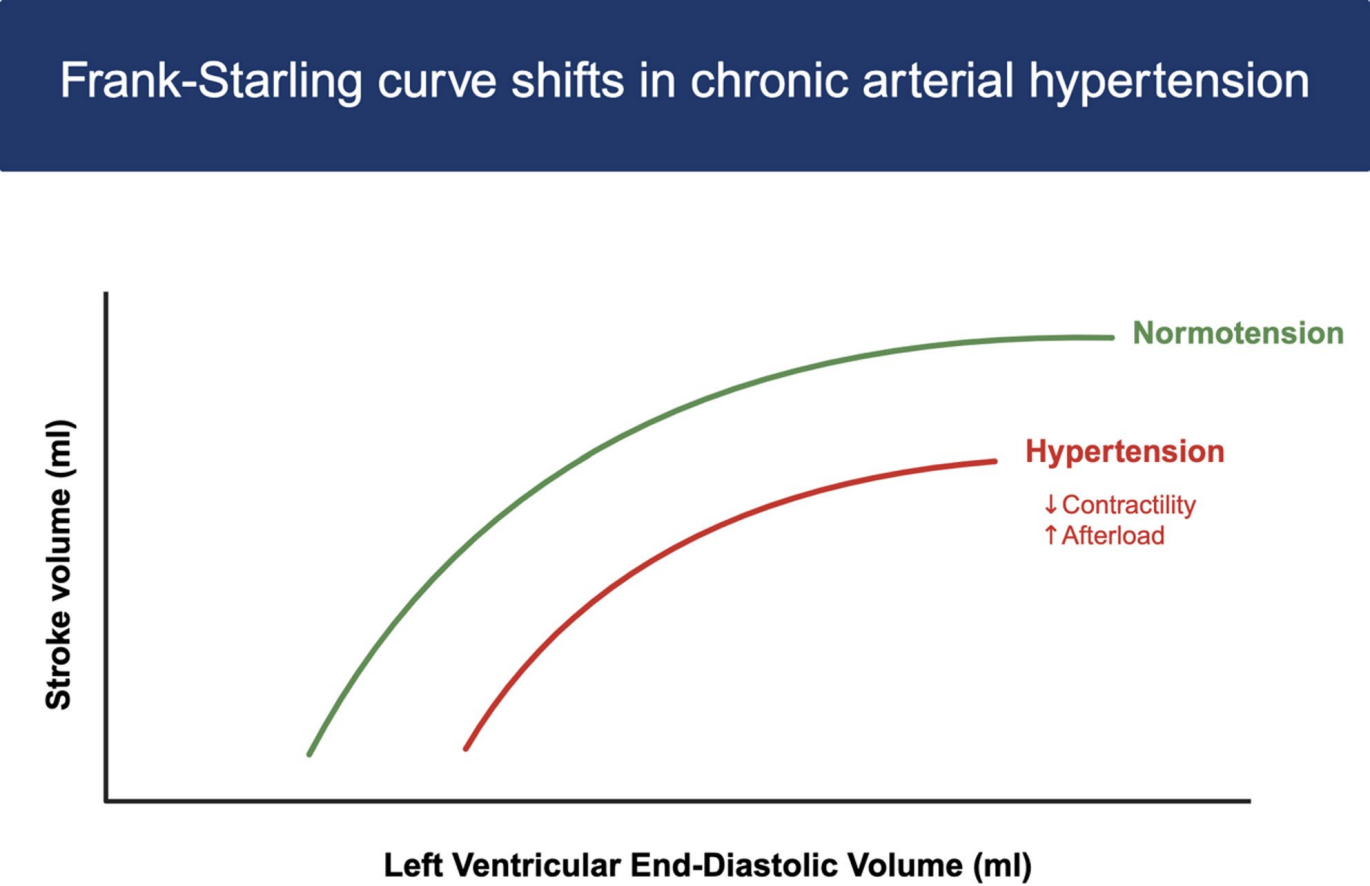
Frank-Starling curve shifts in chronic arterial hypertension

Sustained arterial hypertension imposes chronic pressure overload, increasing LV afterload and wall stress, requiring the myocardium to generate higher systolic force to maintain stroke volume. Neurohormonal activation including sympathetic and renin–angiotensin–aldosterone pathways support early compensation. However, longer-term signalling contributes to maladaptive myocardial changes with altered energetics and adverse interstitial remodelling that can impair systolic function and reduce contractile reserve even when resting output appears preserved [10]. The Frank–Starling mechanism explains how greater preload augments stroke volume through the length–tension relationship. In hypertension, increased afterload means that for any given preload the ventricle achieves less fibre shortening and ejection, so maintaining stroke volume often requires a higher operating preload and greater contractile effort. This concept is illustrated in Fig. 3, where the operating point shifts and the curve is functionally “depressed” by increased afterload and reduced ventricular-arterial coupling, despite preserved resting stroke volume [50, 51].

Left ventricular ejection fraction (LVEF) remains the most widely reported descriptor of systolic function. However, it is a measure of change in LV cavity volume rather than a direct measure of myocardial contractility. In hypertension, LVEF can remain normal, and may appear supranormal in some patients, despite impaired myocardial shortening and reduced contractile reserve. Mid-wall mechanics provide additional insight with the mid-wall stress-shortening approach described by de Simone and colleagues (1994) demonstrating that apparently preserved endocardial shortening can mask depressed mid-wall function in hypertension and subsequent work linked impaired mid-wall fractional shortening to adverse phenotypes and outcomes [52, 53]. Speckle-tracking echocardiography has therefore become central to detecting subclinical systolic dysfunction. Global longitudinal strain (GLS) is especially informative as longitudinal fibres are vulnerable early and GLS may decline before changes in LVEF. In a meta-analysis, Tadic and colleagues (2021) showed consistent impairment of strain parameters in hypertensive individuals compared with controls, supporting GLS as a complementary marker of systolic dysfunction [54]. However, GLS should be interpreted cautiously in hypertension. Strain is load dependent and may worsen acutely with increased afterload even when intrinsic myocardial contractility is unchanged, so contemporaneous BP and haemodynamic context are important when comparing values between studies or over time [55]. In addition, despite substantial standardisation efforts, inter-vendor differences and differences in post-processing methodology persist, and test-retest reproducibility, although improving, is not uniform across platforms [56]. There are also no universally accepted hypertension-specific GLS thresholds, meaning that absolute values should be interpreted in the context of image quality, platform, loading conditions and serial change rather than as a standalone diagnostic cut-off [54]. When assessing functional reserve, deformation-based approaches can also be extended to afterload-aware metrics such as non-invasive myocardial work (pressure–strain analysis), introduced by Russell and colleagues (2012) which integrates contemporaneous BP with strain to better reflect systolic performance under increased afterload [57]. Myocardial work is therefore an appealing extension of strain imaging in hypertension, but at present it remains an emerging adjunct rather than a routine standard, with broader implementation limited by the need for further standardisation, wider availability and outcome-linked validation in hypertensive populations [55].

### Diastolic function and filling pressures

LV diastolic dysfunction is often one of the earliest functional abnormalities in HHD, reflecting impaired active relaxation and reduced chamber compliance arising from chronic pressure loading and myocardial remodelling [58]. Echocardiography provides the principal non-invasive framework for characterising diastolic physiology and estimating filling pressures. Current BSE and ASE/EACVI recommendations advocate an integrated approach incorporating transmitral Doppler inflow (E and A velocities, E/A ratio, deceleration time), tissue Doppler annular velocities (e′), and supportive indices such as E/e′, tricuspid regurgitation velocity and indexed LA volume to infer the likelihood of elevated left atrial pressure and LV filling pressures [38, 59]. However, the diagnostic performance of individual parameters is modest, and no single variable is sufficiently robust to be overinterpreted in isolation. E/e′, LA volume and TR velocity each have recognised limitations and may be influenced by age, rhythm, image quality, coexistent cardiac disease and loading conditions. Accordingly, interpretation should be anchored to the clinical context, BP, heart rate and rhythm at the time of acquisition, with greatest diagnostic confidence when multiple indices are concordant and serial comparisons are made under comparable haemodynamic conditions.

In hypertensive cohorts, echocardiographic diastolic abnormalities have been shown to have prognostic implications. Zhou et al. (2022) reported that guideline-defined LV diastolic dysfunction predicted major adverse cardiovascular events in a community hypertension cohort, while Thom et al. (2025) demonstrated that tissue Doppler indices (lower e′ and higher E/e′) predicted long-term cardiovascular mortality in the ASCOT legacy follow-up [60, 61]. Persistently elevated filling pressures and reduced diastolic reserve contribute to symptoms and the clinical syndrome of heart failure with preserved ejection fraction (HFpEF), in which exertional rises in filling pressures are a key mechanism despite preserved ejection fraction [62, 58]. Where resting indices are equivocal, but symptoms suggest HFpEF, guideline-based approaches support evaluation under physiological stress to identify exercise-induced elevation of filling pressures [38, 59]. These observations reinforce the need for an integrated rather than parameter-by-parameter interpretation of diastolic function in hypertension.

### Atrial remodelling and function

In arterial hypertension, the left atrium (LA) serves as a marker of cumulative diastolic burden, with sustained or intermittent elevation of LV filling pressures driving progressive LA dilatation, most accurately quantified by indexed LA volume rather than linear diameter [13]. LA enlargement is therefore a marker of chronic pressure load once alternative causes (notably significant mitral valve disease and persistent atrial arrhythmia) are considered. Functionally, LA function is described in three phases: reservoir (during ventricular systole), conduit (passive emptying in early diastole) and contractile (active emptying in late diastole) [63]. In early hypertension and diastolic dysfunction, reduced early diastolic emptying may be offset by augmented atrial contraction, whereas longer-standing disease is characterised by fibrosis, impaired reservoir function and enlargement, creating a substrate for atrial fibrillation (AF) [10].

LA remodelling has important prognostic implications. Imaging recommendations highlight LA enlargement as an independent marker of adverse outcome and a structural correlate of chronic diastolic dysfunction [13, 17]. In a community population of 356 patients with suspected heart failure, indexed LA volume independently predicted mortality beyond clinical and other echocardiographic parameters, supporting its role as a robust risk marker [64]. Beyond size, speckle-tracking–derived LA strain (particularly reservoir strain) provides a quantitative measure of atrial function and may refine interpretation of diastolic physiology when conventional indices are borderline or discordant [65]. In hypertension, impaired LA strain may precede dilatation and has been linked to HMOD. In a prospective cohort of 135 participants including controls, Seçkin et al. (2025) reported lower LA reservoir strain in non-dipper hypertension (characterised by insufficient nocturnal BP reduction) was associated with hypertensive nephropathy defined by microalbuminuria [66]. Load- and rhythm-dependence must also be considered as AF alters atrial mechanics (loss of effective contractile phase, irregular cycle lengths) and Doppler-derived indices, so acquisition and interpretation should be contextualised to rhythm and loading conditions at the time of assessment [13].

### Right ventricular and pulmonary circulation assessment

Although arterial hypertension is classically viewed as a left-sided disorder, growing evidence suggests that right ventricular (RV) remodelling with hypertrophy and dysfunction can occur in individuals with hypertension, most often as a downstream consequence of raised left-sided filling pressures, pulmonary venous hypertension and adverse ventricular–arterial coupling [67]. There is also shared myocardial fibrosis, microvascular dysfunction and cardiometabolic comorbidity [68, 69]. These RV changes are frequently subclinical and may be detected only with comprehensive echocardiographic assessment, reinforcing the need to interpret RV findings in the context of overall haemodynamic and symptom burden.

Estimation of pulmonary pressures is central to this evaluation. Echocardiography provides a non-invasive estimation of pulmonary hypertension using tricuspid regurgitation velocity supported by right-heart morphology and functional signs, while also allowing assessment of RV adaptation that strongly influences prognosis across cardiopulmonary disease [70]. In arterial hypertension, mildly elevated pulmonary pressures are often driven by left heart disease (diastolic dysfunction and LA remodelling) and therefore should be integrated with contemporaneous loading conditions and clinical context rather than interpreted as isolated pulmonary vascular disease [70].

Beyond conventional measures such as tricuspid annular plane systolic excursion (TAPSE), fractional area change and tissue Doppler S′ velocity, myocardial deformation imaging has provided additional insight into subclinical right ventricular dysfunction in hypertension. Speckle-tracking–derived RV free-wall longitudinal strain may detect subtle impairment when conventional indices remain within normal limits, although the evidence in hypertension is limited and largely observational. In hypertensive cohorts, impaired RV strain has been associated with reduced functional capacity and adverse atrioventricular coupling, suggesting that right-heart involvement may contribute to exercise intolerance beyond left-sided abnormalities alone [67]. Tadic and colleagues (2014) reported associations between right atrial and RV mechanics and functional capacity in hypertensive patients, supporting the concept of integrated atrioventricular assessment [71]. Cardiac magnetic resonance–based strain analyses have further demonstrated biventricular myocardial deformation abnormalities in hypertensive heart disease, supporting the concept that hypertensive remodelling is not exclusively left-sided [72]. Contemporary echocardiographic guidance recognises deformation imaging as a complementary parameter in comprehensive right-heart assessment, particularly when conventional parameters are equivocal or when pulmonary pressures are borderline [70]. Although load-dependence and vendor variability must be considered, RV strain may enhance phenotyping of hypertension-mediated cardiac damage and refine understanding of right-heart adaptation in selected patients.

Ventricular interdependence underpins the physiological relationship between pulmonary pressures, RV function and LV compliance. The ventricles share the interventricular septum and are constrained by the pericardium [73]. Therefore, increases in RV pressure or volume loading can shift the septum and impair LV filling and stroke volume, while elevated LV filling pressures increase pulmonary venous pressure and RV afterload. Practically, this means RV and pulmonary pressure indices should be interpreted alongside BP, rhythm and volume status at the time of acquisition [70].

### Valvular heart disease

Arterial hypertension interacts bidirectionally with valvular heart disease (VHD) as it may contribute to valve lesion development, while established valve disease amplifies adverse ventricular loading and remodelling. In a large UK electronic health record cohort (5.4 million adults), long-term exposure to higher BP across its spectrum was associated with increased risk of both aortic stenosis and aortic regurgitation, supporting elevated BP as a potentially modifiable risk factor for aortic valve disease [74]. Similarly, in a parallel UK cohort study (5.5 million adults), higher long-term BP exposure was associated with increased risk of incident mitral regurgitation (primary and secondary), suggesting a role for BP control in prevention and disease trajectory [16].

From a haemodynamic perspective, hypertension alters loading conditions and therefore the echocardiographic assessment of valve lesions. In aortic stenosis, elevated BP increases afterload and may reduce forward stroke volume, therefore potentially reducing measured transvalvular gradients such that stenosis severity may be partially masked [75]. In regurgitant lesions, BP influences the driving pressure and regurgitant volume. Uncontrolled hypertension can exacerbate aortic regurgitation by increasing the diastolic pressure gradient and can augment functional mitral regurgitation by increasing LV systolic pressure and mechanical stress on the mitral apparatus [76]. For these reasons, contemporaneous BP measurement is essential when interpreting Doppler haemodynamics and regurgitation severity and repeat assessment after optimisation of BP may be clinically informative where findings and symptoms appear discordant.

Hypertension also impacts myocardial performance in VHD through combined pressure/volume loading, impaired ventricular–arterial coupling and increased myocardial oxygen demand. In aortic stenosis, superimposed hypertension can worsen afterload mismatch and accelerate hypertrophy, fibrosis and diastolic dysfunction, increasing the likelihood of symptoms at a given valve severity and complicating attribution of breathlessness or reduced exercise capacity [75]. In mitral regurgitation, persistent afterload excess may promote LV remodelling and increase regurgitant volume, potentially worsening prognosis and contributing to the transition from compensated to decompensated disease [76]. Practically, integrating the valve lesion with measured BP and indices of myocardial performance provides a more physiologically coherent assessment than valve metrics alone.

### Assessment of the aorta

Hypertension is an important contributor to aortopathy and evaluation, when echocardiography is undertaken to evaluate HMOD, should include assessment of the aorta [13]. Coarctation of the aorta is a well-recognised structural cause of hypertension and LVH that may be missed clinically, especially in younger adults. Echocardiography is central to raising and supporting this diagnosis [77]. Accordingly, a dedicated 2D, colour and spectral Doppler assessment of the distal arch and upper descending thoracic aorta (suprasternal), with careful interrogation for a focal narrowing and Doppler evidence of obstruction should be performed. In most patients TTE allows reliable measurement of the aortic root and proximal ascending aorta and cross-sectional imaging with CT or CMR are used when echocardiographic windows are limited or when comprehensive thoracic aortic mapping is required. When aortic dilatation is identified, surveillance strategy should be individualised according to diameter, growth rate and clinical context (e.g. bicuspid aortic valve, family history, syndromic aortopathy and post-intervention status), with selection of modality guided by the segment requiring follow-up and image quality [78].

Chronic aortic regurgitation (AR) is frequently associated with arterial hypertension and wide pulse pressure due to increased stroke volume and diastolic reflux, which can worsen LV remodelling and prognosis [79]. The haemodynamic consequences of AR are a combination of volume and pressure overload, resulting in elevated systolic BP and reduced diastolic BP, manifesting as a wide pulse pressure contributing to progressive LV dilatation and dysfunction. Long-term exposure to elevated BP, particularly increased systolic and pulse pressure, is linked to higher risk of developing AR. Hypertension contributes to secondary AR by aortic root dilatation and is associated with a higher prevalence of mild AR in hypertensive populations [80]. The BSE practical guideline on AR emphasises an integrated multiparametric approach and recommends documenting BP at the time of study to support accurate grading and clinical interpretation [81].

### Transoesophageal echocardiography considerations

Transoesophageal echocardiography (TOE) is not routinely indicated in arterial hypertension, although it may be required when TTE is non-diagnostic or when higher resolution assessment of valves, LA appendage, endocarditis complications or the thoracic aorta is needed [82]. As TOE is semi-invasive, it frequently requires conscious sedation and a structured pre-assessment and continuous physiological monitoring, including automated BP measurement, ECG and pulse oximetry is recommended throughout the study [83, 84]. In most UK services, midazolam is the first-line sedative for TOE and should be titrated to the minimum effective dose with adequate time allowed between doses to monitor effect. Midazolam can, however, produce clinically relevant haemodynamic effects. Midazolam has been associated with a decrease in mean arterial pressure and systemic vascular resistance, resulting in potential falls in cardiac output and stroke volume, particularly with higher or rapidly administered intravenous doses or in vulnerable patients.[85, 86] These effects will be further compounded by the patient’s low volume state related to pre-procedure fasting and limited oral intake. Therefore, hypertensive patients, especially those receiving concomitant antihypertensive therapy, older patients, autonomic dysfunction and obstructive sleep apnoea should be monitored closely for sedation-related hypotension with readiness to adjust dosing and provide appropriate support [87]. For interpretation, haemodynamic findings and Doppler-based assessments should be contextualised to loading conditions at the time of acquisition.

### Future directions

The prevention paradigm emphasised by contemporary hypertension guidelines is urgent given the scale of the problem. An estimated 1.4 billion adults aged 30–79 years worldwide had hypertension in 2024 with only a minority having their BP controlled [1]. In parallel, the global obesity crisis with over 890 million adults living with obesity in 2022 is accelerating the incidence of hypertension and cardiometabolic disease, particularly in younger populations [88]. In this context, a key future challenge is designing hypertension pathways that integrate lifestyle and weight-focused prevention with targeted imaging and specialist input, rather than defaulting to resource-intensive investigations for all. Public and patient-facing initiatives will remain central to this strategy. Blood Pressure UK is the UK’s leading patient-focused charity dedicated to improving BP awareness and control, and its campaigns aim to identify the “missing millions” living with undiagnosed hypertension through opportunistic and home BP checks [89]. In 2006, the European Society of Hypertension (ESH) developed the Hypertension Excellence Centre programme, to recognise clinical institutions providing the highest level of care for patients with hypertension, with over 120 centres certified to date [90]. Complementing this initiative, the British and Irish Hypertension Society (BIHS) has described the rationale for specialist referral centres in the UK and is developing a Hypertension Specialist Accreditation Scheme to recognise both centres and individual clinicians with advanced expertise in hypertension [91]. Such models could support standardised pathways for complex hypertension, improve access to work-up and provide a platform for training, audit and multicentre research in HHD.

Beyond service development, the next phase of progress in HHD will depend on coordinated research priorities. The BIHS, in collaboration with the British Heart Foundation Clinical Research Collaborative (BHF CRC), has recently articulated national research priorities in hypertension, emphasising earlier-life disease, precision phenotyping, integration of digital health tools, and improved risk prediction beyond clinic BP alone [92]. These priorities align closely with the need to better characterise hypertension-mediated cardiac damage in younger adults and to understand trajectories of structural remodelling over the life course. In this context, the forthcoming joint BIHS–UK Kidney Association (UKKA) RaDaR registry initiative for prospective data collection in individuals with hypertension under the age of 40 represents a significant opportunity [93, 94]. Systematic capture of imaging, biochemical and longitudinal outcome data in younger populations could clarify the natural history of early hypertensive cardiac remodelling, define thresholds for intervention, and determine whether echocardiographic markers add value in this group where lifetime risk is high but short-term event risk is low. Embedding standardised echocardiographic datasets within such registries may help move the field from descriptive phenotyping to outcome-linked risk modelling.

Echocardiography is likely to have an expanding role in cardiovascular risk stratification by identifying cardiac structural and functional manifestations that can refine risk beyond clinic BP alone and support decisions about treatment intensity. Where echocardiography is indicated, it is reasonable to advocate that the first study should be a comprehensive baseline TTE, to provide robust phenotyping and a reference for future comparison. In the same way that the BSE applies specific protocols for the ongoing assessment of haemodynamic compromise in pericardial effusions, or for serial assessment of ejection fraction and GLS in cardio-oncology, a structured but streamlined approach to baseline assessment in hypertension could improve standardisation and clinical utility [95]. However, repeat echocardiography should be individualised and financially sustainable. In most patients, confirming real-world BP burden with 24-hour ambulatory or home BP monitoring should be prioritised when interpreting cardiac changes and judging response, while the ECG remains the simplest scalable initial tool despite its limitations. Standardisation of reporting will become increasingly important if echocardiography is to inform preventive care at scale. The inclusion of BP at the time of the study should be routine as afterload influences interpretation of echo parameters. Artificial intelligence (AI) is likely to accelerate reproducible quantification and workflow efficiency, and implementation must be anchored in validation, governance and interoperability. Multimodality integration should be used selectively where it adds clear diagnostic value, particularly for suspected secondary causes of hypertension, where CT/MR techniques can support evaluation of renovascular disease, coarctation and adrenal pathology within specialist pathways, including young-onset hypertension [10].

## Conclusion

Echocardiography has a central role in contemporary hypertension care, not as a universal screening test but as a targeted investigation to detect and phenotype cardiac HMOD when results will change risk stratification or management. Echocardiography provides insight that complements BP measurement, and its interpretation should be anchored to haemodynamic context with contemporaneous BP documented in reports. Future priorities may include embedding echocardiography within prevention-focused and financially sustainable pathways, individualising repeat imaging based on risk profile and BP burden, and exploring how automation and AI could support reproducibility and workflow. Collaboration between imaging and hypertension stakeholders, supported by specialist services and practical consensus guidance, is likely to be key to standardising care and advancing research in HHD.

## Data Availability

No datasets were generated or analysed during the current study.
